# Hypertension attenuates COVID-19 vaccine protection in elderly patients: a retrospective cohort study

**DOI:** 10.3389/fimmu.2025.1612205

**Published:** 2025-07-28

**Authors:** Zhen Yuan, Iong Fong Wong, Ren-He Xu, Xiao Zhan Zhang, Chon Lok Lei

**Affiliations:** ^1^ Faculty of Health Sciences, University of Macau, Macao, Macao SAR, China; ^2^ Pneumology Department, Kiang Wu Hospital, Macao, Macao SAR, China

**Keywords:** COVID-19, vaccination, prognosis, elderly patients, comorbidities

## Abstract

The COVID-19 pandemic has severely impacted elderly populations, particularly those with comorbidities. This study evaluated the effects of COVID-19 vaccination on 193 hospitalized elderly patients (≥ 60 years) in Macao. Vaccination was suggestively associated with a 2.3-fold higher likelihood of prognostic improvement (adjusted OR = 2.3, 95% CI: 0.980-5.940, *P* = 0.065), while hypertension significantly reduced the improvement rate by 67.5% (*P* = 0.039). Vaccinated patients also exhibited lower Modified Early Warning Scores and reduced mortality. These findings underscore the protective role of vaccination in improving prognosis among high-risk elderly patients and highlight the need for tailored strategies for those with comorbidities.

## Graphical Abstract

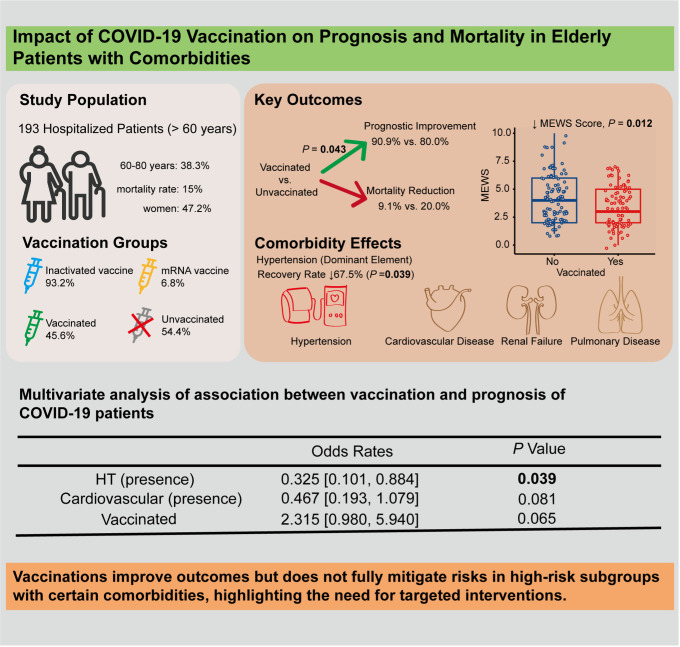

## Introduction

The COVID-19 can affect individuals of all ages, especially those with weakened immune systems and comorbidities, increasing the risk of severe disease and mortality. Since the introduction of COVID-19 vaccines, vaccination has been shown to significantly reduce rates of infection, severe disease, and mortality (1).

Different types of vaccines, such as mRNA vaccines (Pfizer-BioNTech) and inactivated vaccines (Sinovac, Sinopharm) (2), have varying immunogenicity and efficacy, especially in older adults (3). Individuals aged 65 and older, as well as those with at least one underlying medical condition, remain at elevated risk for severe outcomes from COVID-19 even after completing primary vaccination (4). This study evaluates the clinical impact of vaccination on elderly hospitalized patients with comorbidities, focusing on prognosis improvement and mortality reduction to inform public health strategies.

## Method

### Study population

This retrospective study included patients aged 60 years and older who were hospitalized at Kiang Wu hospital in Macao between December 15, 2022, and March 15, 2023.

### Inclusion criteria

(1) Residents of Macao who were aged 60 years or older. (2) Laboratory-confirmed COVID-19, defined as either a positive result from a nasopharyngeal swab rapid antigen test or a nucleic acid test with a cycle threshold value of less than 39. (3) Presence or absence of fever, respiratory symptoms, gastrointestinal symptoms, or other clinical manifestations of COVID-19.

### Exclusion criteria

(1) Incomplete clinical data. (2) Hospitalization for less than 24 hours.

### Diagnostic and treatment protocols

The diagnosis, severity classification, and treatment protocols followed the guidelines outlined in the Ninth Edition of the Chinese Medical Association COVID-19 Diagnosis and Treatment Protocol. Serum antibody for SARS-CoV-2 S (spike, RBD) was quantified using the Elecsys^®^ Anti-SARS-CoV-2 S kit (Roche) via electrochemiluminescence immunoassay, following the manufacturer’s instructions. Results were reported in U/mL, with< 0.80 U/mL considered negative and ≥ 0.80 U/mL positive.

### Statistical analysis

The study population was stratified by age (60–80 years and >80 years) and vaccination status (unvaccinated or vaccinated). Patients with mixed vaccination types were excluded to minimize confounding. Vaccine type (e.g., mRNA, inactivated), dose count (1, 2, 3, 4), and concentrations of anti-S protein antibodies categorized into three levels (<0.8, 0.8–25000, >25000). The underlying medical conditions include hypertension (HT), diabetes mellitus (DM), tumors, renal failure, cardiovascular disease, and pulmonary disease. Prognosis categorized as either “improvement” or “mortality” alongside Modified Early Warning Score (MEWS) and chest X-ray (CXR) scores on hospital admission.

For the baseline analysis, categorical variables were described as counts and percentages. Fisher’s Exact Test was used to calculate the *P*-value for each dichotomous variable. To enhance the reliability of the results, we further employed binomial logistic regression to adjust for confounders Equations 1, 2:


(1) Y ∼ B(m,p)

(2) $$g\left(p\right)=\beta_{0}+\beta_{1}x_{1}+\;\ldots +\;\beta_{k}x_{k}$$

(3) $$logit\left(p\right)=In\left(\frac{p}{1-p}\right)$$

where *m* is the total patients, *B* is the binomial distribution, *Y* is the number of patients with improved prognosis or anti-S antibody levels out of *m* patients, *g*(*p*) is improvement prognostic status, and *p* is the probability of patients with improved prognosis under the given conditions of *x*_1_,…,*x_k_*, where *x_i_* are different dichotomous variables: gender, age, vaccinated or not, cardiovascular disease, renal failure, HT, DM, tumor, other disease, and pulmonary disease, with *k* = 10. Some dichotomous variables (in the data) were not included in the construction of the model due to the assumption of independence of variables, e.g., the number of doses and “whether or not vaccinated” are related to the type of vaccine and the concentrations of anti-S protein antibodies. Using Equations 3 to perform a confounding factor analysis, *p*/(1 - *p*) is the ratio of the probability of success to the probability of failure and is called the OR. We then chose the final independent variable (HT, DM and type of vaccine) by the Akaike information criterion (AIC) in a stepwise algorithm (the direction is both ways), and the maximum likelihood estimate is used to calculate the *P*-value.

We conducted a generalized linear model analysis using a Poisson distribution to evaluate CXR scores and MEWS, respectively.


Y ∼ Poisson(λ)

$$g\left(\lambda \right)=\beta_{0}\;+\;\beta_{1}\;x_{1}\;+\;\ldots \;+\;\beta_{n}\;x_{n}$$

Where *Y* is the CXR or MEWS, where *x_i_* are different dichotomous variables: gender, age, vaccinated or not, cardiovascular disease, renal failure, HT, DM, tumor, other disease, and pulmonary disease, with *n* = 10. The subsequent steps are similar to prognostic analysis. All analyses were performed in R (version 4.4.1), and a *P*-value of< 0.05 was considered statistically significant.

## Results

A total of 193 hospitalized COVID-19 elderly patients in Macao were included in this study (**Tables 1**, **2**), comprising 91 women (47.2%). The median age was 83 years, with 119 patients (61.7%) aged > 80 years and 74 patients (38.3%) aged between 60 and 80 years. The overall mortality rate in this cohort was 15% (29/193). Among the participants, 45.6% (88/193) were vaccinated, including 82 patients (93.2%) who received inactivated vaccines and 6 patients (6.8%) who received mRNA vaccines. Vaccinated patients had a markedly lower mortality rate (9.1%, 8/88) compared to unvaccinated patients (20.0%, 21/105). Comorbidities were prevalent (**Table 2**): hypertension (65.3%, 126/193), cardiovascular disease (48.7%, 94/193), diabetes mellitus (34.2%, 66/193), renal failure (20.7%, 40/193), pulmonary disease (17.6%, 34/193), and tumors (16.1%, 31/193).

**Table 1:** Baseline characteristics.

Subgroup	Mortality N=29	Improvement N=164	*P* value
Gender			0.549
Female	12 (41.4%)	79 (48.2%)	
Male	17 (58.6%)	85 (51.8%)	
Age			0.415
>80	20 (69.0%)	99 (60.4%)	
60-80	9 (31.0%)	65 (39.6%)	
Vaccine			0.043*
No	21 (72.4%)	84 (51.2%)	
Yes	8 (27.6%)	80 (48.8%)	
Vaccine type			0.067
inactivated	7 (24.1%)	75 (45.7%)	
mRNA	1 (3.4%)	5 (3.0%)	
none	21 (72.4%)	84 (51.2%)	
Concentrations of anti-S protein antibodies			0.276
>25000	1 (3.4%)	20 (12.2%)	
0.8-25000	19 (65.5%)	107 (65.2%)	
<0.8	9 (31.0%)	37 (22.6%)	
Number of doses			0.030*
0	21 (72.4%)	84 (51.2%)	
1	0 (0.00%)	28 (17.1%)	
2	4 (13.8%)	22 (13.4%)	
3	3 (10.3%)	28 (17.1%)	
4	1 (3.4%)	2 (1.2%)	

*P*-value is based on Fisher’s Exact Test. *P< 0.05

**Table 2:** Baseline characteristics with underlying medical conditions.

Subgroup	Mortality N=29	Improvement N=164	*P* value
Pulmonary disease			0.604
No	23 (79.3%)	136 (82.9%)	
Yes	6 (20.7%)	28 (17.1%)	
Cardiovascular			0.069
No	10 (34.5%)	89 (54.3%)	
Yes	19 (65.5%)	75 (45.7%)	
Renal failure			1.000
No	23 (79.3%)	130 (79.3%)	
Yes	6 (20.7%)	34 (20.7%)	
Tumor			0.424
No	23 (79.3%)	139 (84.8%)	
Yes	6 (20.7%)	25 (15.2%)	
DM			0.833
No	20 (69.0%)	107 (65.2%)	
Yes	9 (31.0%)	57 (34.8%)	
Other disease			0.774
No	15 (51.7%)	75 (45.7%)	
Yes	14 (48.3%)	87 (53.0%)	
Unknown	0 (0%)	2 (1.3%)	
HT			0.035*
No	5 (17.2%)	62 (37.8%)	
Yes	24 (82.8%)	102 (62.2%)	

*P*-value is based on Fisher’s Exact Test. *P< 0.05

Vaccinated patients had significantly better prognostic outcomes compared to unvaccinated patients. Among the vaccinated group, 90.9% (80/88) showed improvement, compared to 80.0% (84/105) in the unvaccinated group (*P* = 0.043) (**Table 1**). Multivariate logistic regression analysis indicated that vaccination was suggestively associated with a 2.3-fold improvement in prognosis (95% CI: 0.980–5.940, *P* = 0.065) (graphical abstract table), whereas hypertensive patients were 67.5% less likely to improve than non-hypertensive patients (OR = 0.325, 95% CI: 0.101–0.884, *P* = 0.039) (**Table 3**).

**Table 3:** Multivariate logistic regression for prognostic improvement.

Subgroup	Rate	*P* value
Age 60-80	1.136 [0.462,2.944]	0.785
Vaccine (yes)	2.3 [0.980,5.940]	0.065
HT (presence)	0.325 [0.101,0.884]	0.039*

*P*-value is based on Wald Test. *, *P* < 0.05. Values in parentheses indicate 95% confidence intervals.

Severe COVID-19 symptoms are correlated with higher adjusted Modified Early Warning Scores (MEWS) (5). The chest X-ray (CXR) scoring system has since been applied to quantify pulmonary damage in COVID-19 patient (6). Through multivariate logistic regression, vaccinated patients had significantly lower MEWS compared to unvaccinated patients (Adjusted Rate = 0.828, 95% CI: 0.715–0.958, *P* = 0.012). However, no significant difference was observed in CXR scores between the two groups (**Table 4**). Patients with renal failure and pulmonary disease exhibited significantly higher CXR scores (+17.6% and +16.8%, respectively; both *P<* 0.01), but no significant difference in mortality was observed.

**Table 4:** Confounding factor analysis for CXR scores.

Subgroup	Rate	*P* value
Age 60-80	1.156 [1.066,1.254]	< 0.001***
Cardiovascular (presence)	0.927 [0.854,1.006]	0.071
Pulmonary Disease (presence)	1.168 [1.055,1.291]	0.0026**
Renal Failure (presence)	1.176 [1.069,1.292]	< 0.001***
Tumor (presence)	0.837 [0.743,0.941]	0.0032**
DM (presence)	1.097 [1.007,1.195]	0.034*

*P*-value is based on Wald Test. *P< 0.05; **P< 0.01; ***P< 0.001. Values in parentheses indicate 95% confidence intervals.

**Figure 1:**
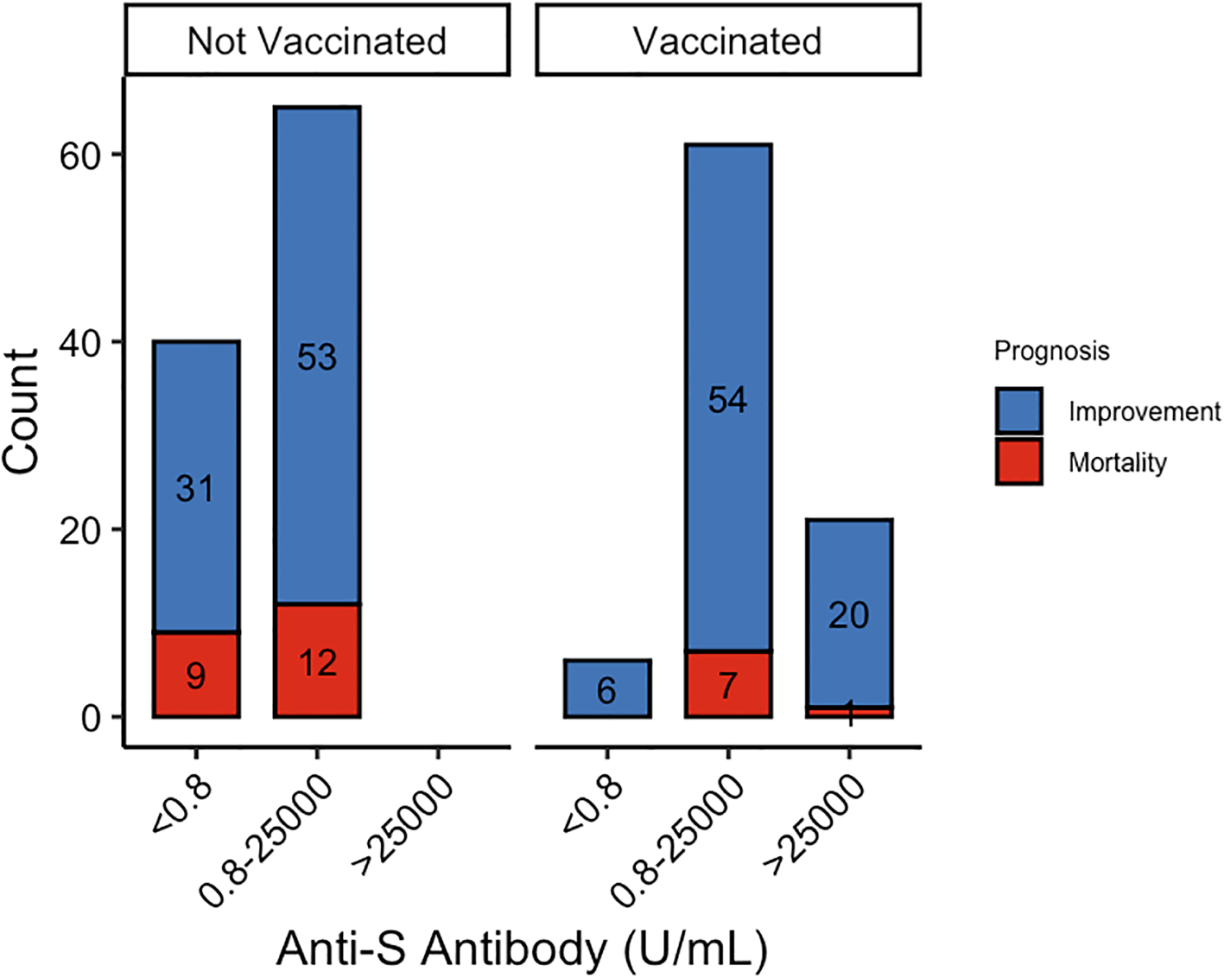
The number of patients with different immunity strength with or without vaccination.

Additionally, vaccinated patients had significantly higher concentrations of anti-S protein antibodies, which were associated with reduced mortality rates (**Figure 1**). We then analyzed the relationship between anti-S antibody levels and clinical outcomes. While anti-S antibody levels were not directly associated with prognosis, patients with antibody concentrations > 25,000 exhibited significantly lower MEWS scores (71.4%, *P* = 0.025) compared to those with lower antibody levels.

## Discussion

This study demonstrates that COVID-19 vaccination, particularly with inactivated vaccines, significantly improves prognosis and reduces mortality in elderly hospitalized patients with comorbidities. Vaccinated patients exhibited a suggestive trend toward improved prognosis, with an adjusted odds ratio of 2.3 and significantly lower MEWS scores. However, comorbidities such as hypertension remained a critical risk factor, reducing the likelihood of recovery by 67.5%. These findings align with previous research (7) highlighting the protective effects of vaccination in high-risk populations while providing novel insights into its specific impact on clinical scores and mortality in elderly patients with multiple comorbidities.

Despite the well-documented protective effects of vaccination, our study highlights persistent challenges associated with comorbidities. Pulmonary disease and renal failure are correlated with worse clinical outcomes and are critical considerations in clinical decision-making. Notably, while vaccinated patients exhibited higher anti-S antibody levels, these levels were not directly correlated with prognosis, suggesting that antibody titers alone may not fully predict clinical outcomes. Instead, neutralizing antibodies play a more direct role in viral clearance and the Sinopharm vaccine, an inactivated whole-virus vaccine, induced a broader antigenic response and higher neutralizing antibody titers than mRNA vaccines, potentially contributing to the lower mortality observed in vaccinated patients (8, 9).

The study’s retrospective design and limited sample size from a single center limit the generalizability of the findings. Future research should involve larger, multi-center cohorts to validate these associations and explore additional risk factors. Additionally, investigating the synergistic effects of vaccination and adjunctive therapies may further optimize treatment strategies for elderly patients with multiple comorbidities.

Our findings align with prior research (10) demonstrating the protective role of COVID-19 vaccines in elderly populations but offer new perspectives by incorporating detailed clinical severity assessments, where we further evaluated MEWS and CXR scores, providing a more granular assessment of disease severity. Vaccination improves outcomes but does not fully mitigate risks in high-risk subgroups with certain comorbidities, highlighting the need for targeted interventions.

## Data Availability

The original contributions presented in the study are included in the article/supplementary material. Further inquiries can be directed to the corresponding authors.
